# The first case of multiple myeloma treated with ASCT followed by Anti-BCMA CAR-T cells using retrovirus vector: A case report

**DOI:** 10.1016/j.heliyon.2024.e36955

**Published:** 2024-08-27

**Authors:** Liqiong Liu, Wenxiang Zhu, Ning Liu, Shiting Gong, Qihong Ma, Huanhuan Zhou, Nan Zhong, Wei Dai, Lijun Zhao, Rui Sun, Jianxun Wang, Yuanyuan Shi, Zhi Guo

**Affiliations:** a Department of Hematology, Huazhong University of Science and Technology Union Shenzhen Hospital, Shenzhen, 518052, China; b Shenzhen Hospital of Traditional Chinese Medicine, Guangzhou University of Chinese Medicine, Shenzhen, 518033, China; c Shenzhen Cell Valley Biomedical Co., LTD, Shenzhen, 518118, China

**Keywords:** Multiple myeloma, BCMA CAR-T, CRS, Retrovirus vector, Case report

## Abstract

Chimeric antigen receptor T (CAR-T)-cell therapy targeting B-cell maturation antigen (BCMA) is currently one of the promising treatment methods for relapsed/refractory multiple myeloma (MM). Herein, this study is a case report on a 41-year-old male patient with MM. Unfortunately, he still developed multidrug-resistant, refractory, and bone marrow suppression after receiving multiline high-intensity chemotherapy. After a detailed evaluation, the physician recommended autologous hematopoietic stem cell transplantation (ASCT) support, followed by sequential immunotherapy with autologous anti- BCMA CAR-T cells. The CAR-T product is a novel anti-BCMA CAR-T based on Retrovirus vectors (RV). It was worth noting that the patient achieved VGPR (very good partial remission) one month after infusion of anti-BCMA CAR-T cells. Recent tests have found that the M protein was no longer detectable and the patient has achieved CR (complete response). Although grade 3 cytokine release syndrome (CRS) appeared, the symptom was well controlled and immune effector cell-associated neurotoxicity syndrome (ICANS) did not occur. This was the first case report of RV prepared anti-BCMA CAR-T cells combined with ASCT for the treatment of MM patient in clinical practice, indicating that the RV-based anti-BCMA-CAR-T cells with ASCT have excellent therapeutic efficacy and high safety in triple-refractory MM patients.

## 1 Introduction

Multiple myeloma (MM), as one of the malignant tumors, is accompanied by the abnormal proliferation of plasma cells in the bone marrow, the production of large amounts of clonal immunoglobulins, and bone destruction. Their unique biological characteristics make it difficult for most patients to escape the adverse outcomes of primary drug resistance or disease recurrence. According to the GLOBOCAN 2020 data [1], the number of new MM patients in China was 114,000 in 2020 and is expected to reach 167,000 in 2024. MM treatment often uses the number of lines of therapy (LOT) to guide the prognosis. Studies have shown that the number of refractory drugs/drug classes is more likely to distinguish homogeneous patients than the use of LOT [2].

Triple-class refractory multiple myeloma (TCR MM) is defined as unresponsiveness to at least one proteasome inhibitor (PI), immunomodulatory drug (IMiDs), or anti-CD38 monoclonal antibody (MoAbs) [3]. However, chimeric antigen receptor T-cell therapy (CAR-T) significantly improved the therapeutic prospects of MM. CAR-T is an effective immunotherapy that eliminates MM cells by activating T cell immunity. In this adoptive cellular immunotherapy, T-cells from patients or healthy people are first genetically engineered in vitro to insert a CAR that can recognize tumor cells and activate T-cells; then, the in vitro expanded CAR-T cells are infused back to attack tumor cells expressing relevant antigens. CAR-T has achieved remarkable results in the treatment of diseases such as acute lymphoblastic leukemia and diffuse large B-cell tumors. MM is one of the most studied hematological tumors for CAR-T cell therapy [4]. A meta-analysis show that, in patients with relapsed/refractory MM (r/rMM) treated with CAR-T, the objective response rate (ORR) was 77 %, the negative rate of minimal residual disease (MRD) was 78 %, and the median progression-free survival (mPFS) was eight months [5]. Grade 3–4 CRS and ICANS occur in 20 % and 15 % of patients, respectively, indicating that the efficacy and safety of CAR-T in the treatment of MM need to be improved [6]. Among them, the B-cell maturation antigen, a target of MM, has become an attractive target for MM treatment because of its overexpression in myeloma cells and limited expression in other tissues [7]. Moreover, two CAR-T products targeting BCMA using lentiviral vectors have been marketed.

In this study, we developed an efficient anti-BCMA CAR-T preparation system based on retroviral vector technology. A patient with triple-refractory MM treated with ASCT followed by anti-BCMA CAR-T cell therapy has also been reported. One month after treatment, the patient had very good partial remission (VGPR) and achieved complete response (CR) after nine months of infusion.

## 2 Methods

**Fig. 1:**
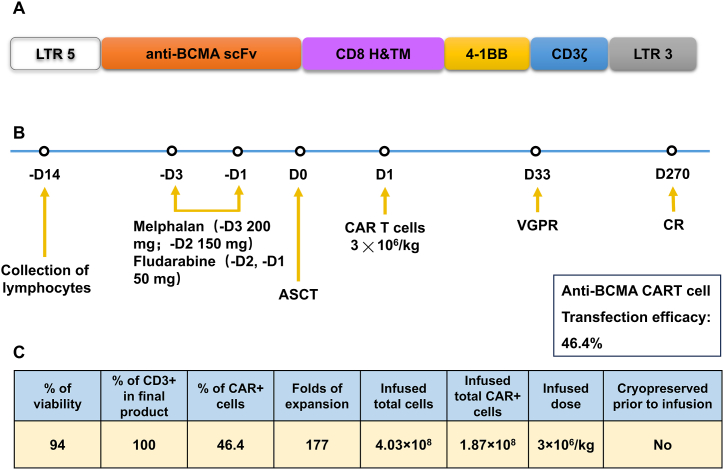
Construction of BCMA-specific CAR and protocol of anti-BCMA CAR-T cell infusions. (A) a schematic diagram of anti-BCMA CAR vector; (B) a protocol of CAR-T infusion. Fludarabine for lymphodepletion. CAR-T cells were infused at a dose of 3 × 10^6^/kg; (C) Characterization of the final anti-BCMA CAR-T cell product.

*CAR construct.* The MFG retroviral vector (RV) was first used to encode an anti-BCMA CAR (Fig. 1A). The anti-human BCMA single-chain variable fragment (scFv) used C11D5.3, where the variable heavy and light chains were separated by a (G4S) 3 linker. The remaining structures were CD8 hinge, transmembrane region, intracellular 4-1BB co-stimulatory domain, and CD3ζ T cell activation domain.

*RV packaging*. The virus production used two cell lines, Phoenix ECO and PG13. By adopting a two-step packaging method, a stable PG13 RV production cell line was formed for virus production [8]. RV was used to transduce activated human T cells.

*CAR-T manufacture*. Firstly, the preparation of CAR-T cells involved isolating autologous peripheral blood mononuclear cells (PBMCs) from the patient's blood. Fresh PBMCs were required to be transported to Shenzhen Cell Valley within 4 hours. The subsequent preparation process were carried out in a C-level clean site. Then, positive selection and enrichment of CD3+T cells were performed using CD3+microbeads. Afterwards, CD3/CD28 antibodies activated CD3+T cells for 2 days. Finally, the activated cells were genetically modified using γ-RV to express anti-BCMA CAR. Flow cytometry (FC) and quantitative polymerase chain reaction (qPCR) were respectively used to detect transduction efficiency and RV copy number. Stimulate with IL-2 cytokines and enrich anti-BCMA CAR-T cells to therapeutic doses. The entire preparation process took about 10 days.

*Cell drug preparation*. After quality control testing and meeting release requirements, the anti-BCMA CAR-T cells that met the therapeutic doses were stored in the appropriate cryogenic storage system. Anti-BCMA CAR-T cells were transported to the treatment hospital of Huazhong University of Science and Technology Union Hospital within 2 hours. Anti BCMA CAR-T cells were injected into the patient's body under strict restrictive conditions. CAR-T cells were administered in a single infusion at a total dose of 3 × 106/kg, containing 1.8 × 108 CAR + T cells. The treatment process was shown in Fig. 1B and C.

*Blood biochemical and pathological measurement.* Monoclonal protein content was detected using serum protein electrophoresis. IgG and Kappa levels were determined by immunofixation electrophoresis. Changes in patients' platelet (PLT), hemoglobin (Hb), white blood cell (WBC), and Neutrophil levels were determined by complete blood cell count (CBC). Serum levels of IL-6, IL-8, and IL-10 were measured by FC according to the manufacturer's protocol for the Human Th1/Th2 detection kit. Immunohistochemistry for IgG, Kappa, BCMA, CD138, and CD38 was performed by incubating the patient's bone marrow smear with the corresponding primary and secondary antibodies. These indexes were measured at the Huazhong University of Science and Technology Union Shenzhen Hospital.

*CAR transgenes copy number measurement.* The expansion and persistence of CAR-T cells were measured by Shenzhen Cell Valley Biomedical Co., Ltd. Briefly, the percentage and absolute number of CAR-T cells were confirmed by staining with fluorescein-labeled monoclonal antibodies. At the same time, leukocytes from peripheral blood were collected using a split red solution for detection by FC. The CAR gene copy number was quantitatively analyzed using qPCR. Genomic DNA was extracted from CAR-T cells or peripheral blood using a genomic extraction kit and amplified on LightCycler® 480. The CAR copy number was detected against the RV sequence of CAR-T cells, which is part of the CAR structure.

### 2.1 Case presentation

A 41-year-old male was admitted to the gastroenterology department of our hospital with diarrhea, and examination results suggested an increase in liver function globulin. After completing the relevant examinations, he was diagnosed with IgG κ MM (DS IIIB, R-ISS I) in December 2022, and the patient had no chronic history of hypertension, coronary heart disease, kidney disease, or diabetes. The diagnostic results were as follows: bone marrow smear showed 5.5 % plasma cells, while immunofixation electrophoresis and immunohistochemistry suggested IgG κ +. Furthermore, immunohistochemical results were positive for BCMA, CD38, and CD138, and serum protein electrophoresis showed that the serum monoclonal protein (M spike) was 70.28 g/L.

**Fig. 2:**
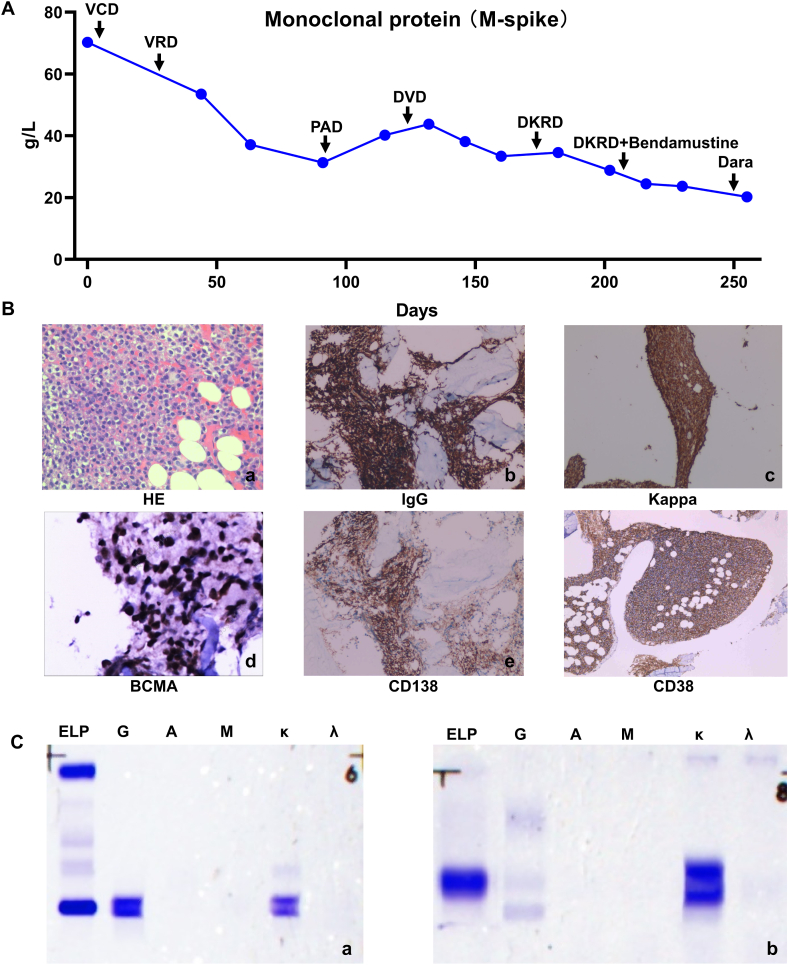
The changes of monoclonal protein (M-spike) and bone marrow aspiration before CAR-T infusion. (A) The levels of monoclonal protein (M-spike) during chemotherapy. DEX: dexamethasone. Dara: Daratumumab.VCD: bortezomib, cyclophosphamide, and DEX. VRD: bortezomib, lenalidomide and DEX. VAD: bortezomib, doxorubicin liposome and DEX. DVD: DEX, bortezomib and Dara. DKRD: Dara, carfilzomib, lenalidomide, and DEX. (B) Bone marrow smear before CAR-T infusion. a: hematoxylin and eosin stain; b–f: immunohistochemistry, IgG positive, Kappa positive, BCMA positive, CD138 positive and CD38 positive. (C) IgG and Kappa were positive in serum and urine. a: immunofixation electrophoresis detection of IgG and Kappa in serum; b: immunofixation electrophoresis detection of IgG and Kappa in urine.

The patient underwent multiple rounds of chemotherapy (Fig. 2A). PR was achieved after 1 week of treatment with VCD (bortezomib, cyclophosphamide, and DEX) and 3 courses of VRD (bortezomib, lenalidomide, and DEX), and the serum monoclonal protein decreased to 31.79 g/L. After 1.5 courses of VAD treatment (bortezomib, doxorubicin liposome, and DEX) and 2 weeks of DVD treatment (DEX, bortezomib, and Dara), the patient’s serum M protein level was 41.25 g/L. Considering disease progression, stem cell apheresis was performed after 1 course of DKD treatment (DEX, carfilzomib and Dara), followed by DKRD (Dara, carfilzomib, lenalidomide, and DEX) and other therapies, the patient's serum M protein decreased to 20.23 g/L. However, the patient showed resistance to multiline antineoplastic drugs and was clinically refractory by detecting relevant indicators through HE, immunohistochemistry, and immunofixation electrophoresis methods (Fig. 2B and C); therefore, CAR-T reinfusion therapy after ASCT was recommended. Fludarabine was used for lymphodepletion on day -2 and day -1, ASCT was performed on day 0, and 50 mL anti-BCMA CAR-T infusion was performed on day 1 (Fig. 1B).

**Fig. 3:**
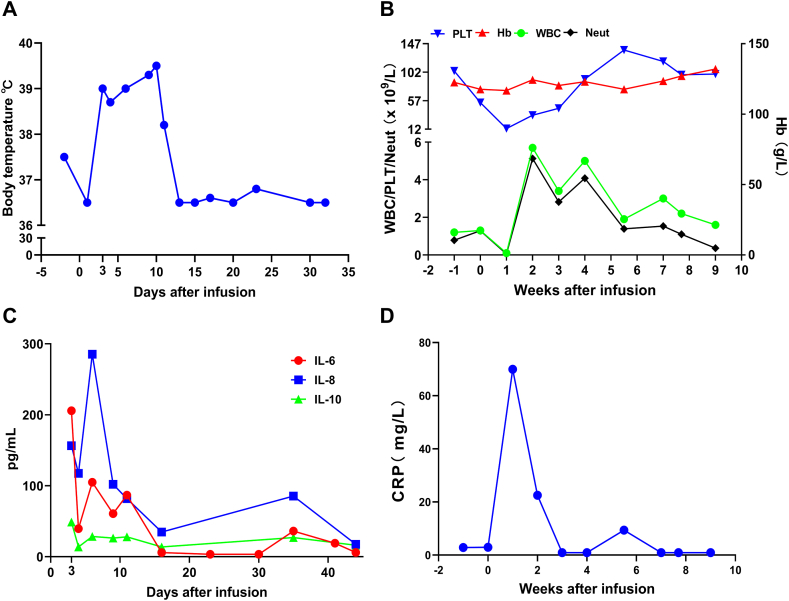
Clinical responses to infusions of anti-BCMA CAR-T cells. (A) Body temperature changes in patient after CAR-T infusion. The body temperature increased to 39.0 °C on the second day and began to decrease on the 11th day. (B) The changes of patient's PLT, Hb, WBC, and Neutrophil levels. (C) Levels of IL-6, IL-8, and IL-10 after CAR-T cell infusion. IL-6, IL-8, and IL-10 increased on the second day and remained at high levels throughout the week after infusion. (D) The changes of patient's CRP. CRP reached its highest level within a week after infusion.

After the infusion of anti-BCMA CAR-T on day 2, the patient developed severe neutropenia with fever and a maximum temperature of 39.5 °C (Fig. 3A). At this time, the patient experienced discomfort such as suffocation, shortness of breath, and decreased partial pressure of oxygen. A computed tomography (CT) scan revealed severe pneumonia in both lungs with type I respiratory failure. The PLT, Hb, WBC, and Neutrophil counts (Fig. 3B), the levels of serum IL-6, IL-8, IL-10 (Fig. 3C), and CRP (Fig. 3D) also increased accordingly. Serum cytokine levels peaked within 1 week after infusion and were 41-, 5- and 10-fold higher than normal levels. The patient was considered to have grade 3 CRS according to the guidelines of the CARTOX Working Group [9], and we performed the necessary screening for cytomegalovirus and Epstein–Barr virus. The RV copy number of BCMA-CARs peaks at 170000 copies/μg.

**Fig. 4:**
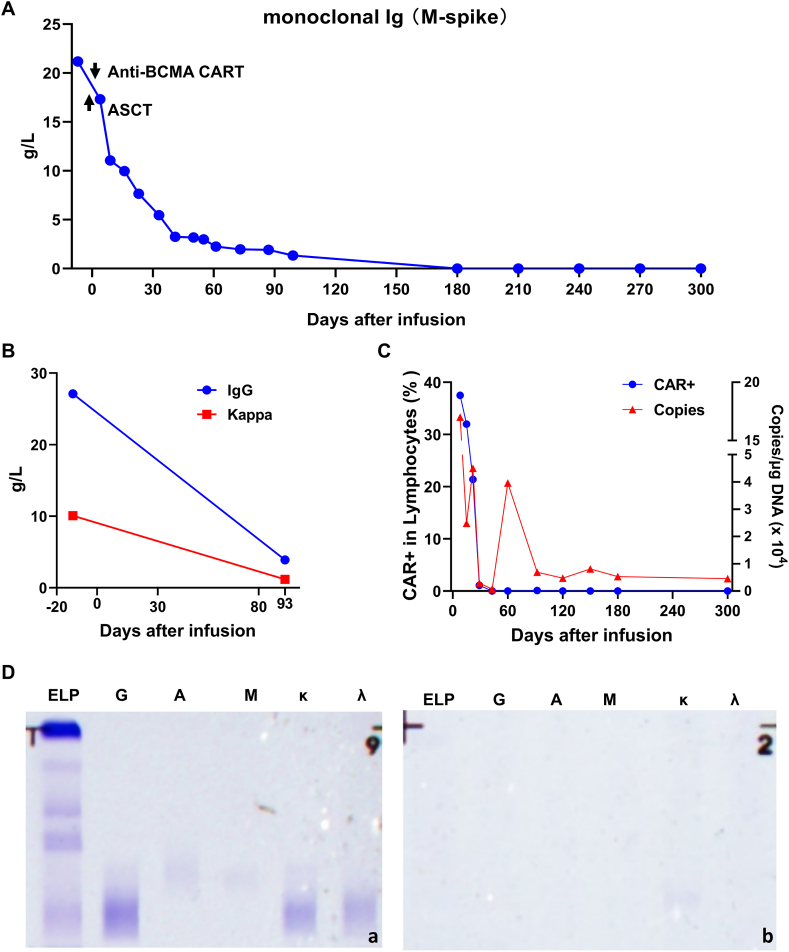
Levels of key indicators after CAR-T infusion. (A) The levels of monoclonal protein (M-spike) after infusion. (B) The changes of IgG and Kappa after infusion. (C) CAR T cell expansion levels detected by FC and CAR DNA copies detected by qPCR. CAR T levels gradually decreased, and CAR DNA copies decreased in a fluctuating manner after infusion. (D) IgG and Kappa were negative in serum and urine. a: immunofixation electrophoresis detection of IgG and Kappa in serum; b: immunofixation electrophoresis detection of IgG and Kappa in urine.

After treatment with antibiotics, gamma globulin, methylprednisolone, and mask oxygen at 6 L/min, the fever was temporarily relieved, the count of PLT, Hb, WBC, Neutrophil, and the levels of CPR and cytokines gradually returned to normal, and the CT scan showed that the bilateral lung infection had been controlled and improved. Although the FC and qPCR results showed that the number of CAR cells and copy number decreased after one month of infusion (Fig. 4C), the content of M protein and IgG continued to decrease gradually (Fig. 4A and B). On day 33, after infusion, the M protein level was 5.46 g/L and continued to decrease. It decreased to 2.26 g/L on day 61 and 1.35 g/L on day 99, indicating that MM was effectively controlled and reached VGPR. Recent tests have found that the M protein is no longer detectable (Fig. 4A) and immunofixation electrophoresis detection of IgG and Kappa are negative in serum and urine (Fig. 4D), the patient has achieved CR.

## 3 Discussion

Currently, a combination of PIs, IMiDs, and anti-CD38 MoAbs is the standard treatment for MM; however, TCR MM has become a clinical problem that cannot be ignored. In addition, ASCT is also considered a standard treatment option for young patients with MM, which is mainly based on response rate and event-free survival considerations; patients can obtain a better quality of life, and the disease can be well controlled without treatment for a considerable period of time. However, in the present case, the patient failed to achieve a synergistic therapeutic effect after multiple rounds of chemotherapy and induction therapy. Finally, multiline antitumor drug resistance has emerged.

BCMA has been identified as a cell surface antigen that is highly specifically expressed on malignant plasma cells, and two commercial CAR-T products have been developed against BCMA, namely idecabtagene vicleucel (ide cel) and ciltacabtagene autoleucel [[10], [11], [12], [13]]. Both agents have been approved for patients with MM who were previously treated with PIs, IMiDs, and anti-CD38 MoAbs. At the same time, most patients in the respective registered clinical trials had also received ASCT. In the KarMMa-1 ide-cel study, 94 % of patients experienced disease progression after ASCT, and in the CARTUDE 1 cilta-cel study, 90 % experienced disease progression after ASCT. Based on the above clinical trials, researchers speculated that relapse after ASCT could be alleviated by early CAR-T cell therapy or that early CAR-T cell therapy might enhance the effect of ASCT treatment, which provides the possibility of ASCT followed by anti-BCMA CAR-T cell therapy in clinical practice [14].

Based on the patient’s current drug resistance status, ASCT alone was not recommended. However, the physician indicated that the patient had a high tumor burden accompanied by a high risk of CRS and ICANS. Even if the tumor could not be effectively eliminated, high-intensity chemotherapy with ASCT could still alleviate the tumor burden. Therefore, the patient was encouraged to receive high-intensity chemotherapy, followed by ASCT support and sequential autologous CAR-T cell-targeted therapy. On the one hand, high-intensity chemotherapy with ASCT is used to reduce the tumor burden and thus reduce the risk of CRS response; on the other hand, the advantage of continuously targeted tumor clearance in vivo is utilized by autologous CAR-T cells. The incidences of CRS and ICNAS in patients with MM treated with anti-BCMA CAR-T are high [6]. In one study, the incidence of ICANS was as high as 41 %, accompanied by different grades of CRS [15]. In this case, we also observed the development of grade 3 CRS; fortunately, the patient improved with treatment at 15 days, and ICANS has not been observed to date. After the infusion of anti-BCMA CAR-T cells, the peak expression of IL6 in the patient was much lower than that reported in the literature [16,17]. Thus, autologous CAR-T cell infusion after ASCT may reduce the incidence of adverse effects.

Regarding efficacy, after 33 days of ASCT and anti-BCMA CAR-T infusion, the patient’s serum M protein content decreased to 5.46 g/L, which was ≥90 % lower than the initial serum M protein content of 70.28 g/L. Based on clinical evaluation, the patient achieved VGPR. Long-term testing of serum M protein expression levels revealed an overall downward trend. After three months of infusion, the serum M protein content of the patient decreased to 1.35 g/L, accompanied by a continuous decrease in serum IgG and Kappa levels; and all were no longer detectable after nine months of infusion. At the same time, after 22 days of anti-BCMA CAR-T cell infusion, the CAR copy number and anti-BCMA CAR-T cell content in peripheral blood remained at a high level, which can effectively and sustainably control the growth of tumor cells. Subsequently, as tumor burden decreased, the proportion of detected CAR-T cells also decreased accordingly. After two months of anti-BCMA CAR-T cell infusion, although CAR-T cells were no longer detected in peripheral blood, the CAR gene could still be detected in peripheral blood. At this time, the anti-BCMA CAR-T cells were mostly memory cells. Once the tumor tends to recur, CAR-T cells can still be activated to monitor and clear tumor cells to avoid tumor progression.

As the key to preparing CAR-T cells, the design of gene transduction vectors is currently dominated by the use of RV and lentiviral vectors [16,17], and the two BCMA CAR-T currently on the market use lentivirus as a vector. However, compared with lentiviral vectors, RV showed advantages in industrial production owing to its ability to produce stable toxigenic cell lines, lower plasmid dosage, lower impurity content, good transduction effect, and high viral vector titer. Thus, it can meet the requirements of high throughput and low cost for CAR-T production while ensuring efficacy and safety. Therefore, in the present study, novel anti-BCMA CAR-T cells were prepared using RV for the first time. It not only achieved significant clinical therapeutic effects but also reflected the feasibility and cost-control advantages of the application of RV in the production of CAR-T cells. Based on the serum M protein content, the patient reached the VGPR level in the first month after treatment. At the same time, there was no abnormal proliferation of CAR-T cells. These results demonstrate the good clinical efficacy and safety of the anti-BCMA CAR T-cell therapy drug.

As a key factor in the preparation of CAR-T cells, the vectors for gene transduction currently mainly involve RV or lentivirus vectors [16,17], and the two BCMA CAR-T currently on the market use lentivirus as a vector. However, compared with the production and use of lentiviral vectors, RV has shown stronger advantages in industrial production processes, such as lower dosage of plasmids, use of stable toxin producing cell lines, lower cost of quality control testing, higher transduction efficiency, and higher viral vector titer. Therefore, while ensuring the safety and efficacy of treatment, it can reduce the production cost of CAR-T and make the production process more efficient. In this study, the CAR-T product is a novel anti-BCMA CAR-T based on RV. RV prepared anti-BCMA CAR-T cells combined with ASCT for the treatment of MM patient in clinical practice was reported, indicating that the RV-based anti-BCMA-CAR-T cells with ASCT treatment had excellent therapeutic efficacy, high safety and cost-control advantages in triple-refractory MM patients.

## 4 Conclusion

In summary, this patient received high-intensity chemotherapy, followed by ASCT support and sequential anti-BCMA CAR T-cell immunotherapy. On one hand, high-intensity chemotherapy with ASCT was used to reduce the tumor burden and thus reduce the risk of CRS response. On the other hand, it reflected the advantage of autologous CAR-T cells in continuous tumor clearance in vivo and validated the advantage of RV application in the preparation of CAR-T cells to control the cost, which also provided an effective clinical product for the treatment of triple refractory MM. This case demonstrated that RV-based anti-BCMA CAR-T cells have good therapeutic effects in patients with triple-refractory MM. This is also the first clinical case of using RV to prepare anti-BCMA CAR-T cells.

## Ethics statement

The study has been approved by the Ethics Committee for Clinical Diagnosis and Treatment Technology Application of Huazhong University of Science and Technology Union Shenzhen Hospital (No:XJS-2012034). The study provided written informed consent forms for patients to participate in the study. Any potentially identifiable images or data in the article could be used for publication after obtaining written informed consent from the participants.

## CRediT authorship contribution statement

**Liqiong Liu:** Supervision, Project administration, Data curation. **Wenxiang Zhu:** Writing – review & editing, Investigation. **Ning Liu:** Visualization, Methodology, Investigation. **Shiting Gong:** Writing – review & editing, Writing – original draft, Data curation. **Qihong Ma:** Writing – review & editing, Writing – original draft, Data curation. **Huanhuan Zhou:** Visualization, Validation. **Nan Zhong:** Visualization, Validation. **Wei Dai:** Visualization, Validation, Conceptualization. **Lijun Zhao:** Writing – review & editing. **Rui Sun:** Writing – review & editing. **Jianxun Wang:** Writing – review & editing, Funding acquisition. **Yuanyuan Shi:** Funding acquisition. **Zhi Guo:** Writing – review & editing, Project administration, Funding acquisition.

## Declaration of competing interest

The authors declare that they have no known competing financial interests or personal relationships that could have appeared to influence the work reported in this paper.
